# Single versus double burr-hole drainage for chronic subdural hematoma: A study of relevant prognostic factors conducted in Pakistan

**DOI:** 10.12669/pjms.35.4.543

**Published:** 2019

**Authors:** Habib Ullah Khan, Khaula Atif, Gholamheidar Teimori Boghsani

**Affiliations:** 1 Habib Ullah Khan, MBBS, FCPS (General Surgery), FCPS (Neurosurgery), National University of Medical Sciences (NUMS), Rawalpindi, Pakistan; 2 Khaula Atif, MBBS, MCPS (Family Medicine), Diploma in Public Health, Diploma in Medical Administration, National University of Medical Sciences (NUMS), Rawalpindi, Pakistan; 3 Gholam-heider Teimori Boghsani, Department of Environmental Health Engineering, School of Health, Torbat Heydariyeh University of Medical Sciences, Torbat Heydariyeh, Iran

**Keywords:** Glasgow Coma Scale, Intracranial Hemorrhage, Seizures, Recurrence, Developing Countries

## Abstract

**Objective:**

To compare the efficacy of single versus double burr-hole for drainage of chronic subdural hematoma, keeping in consideration pertinent demographic, pre and postoperative associations.

**Methods:**

A prospective cohort study carried out in Combined Military Hospital, Multan, (December 2016-August 2018), on adults with diagnosed chronic subdural hematoma (CSDH); being segregated by randomized control trial, non-probability purposive sampling into Group-A and Group-B (who underwent single and double burr-holes for CSDH-drainage respectively). Utilizing SPSS-21, data expressed as frequencies/percentages and mean± standard deviation (SD) and cross-tabulated; p-value <0.05 was taken as significant.

**Results:**

Age and GCS scores were 62±13.694 (range 38-94) and 11.00±3.350 (range 3-15) respectively, males being 40(66.7). Post-operative fatality was Nil, while 8(13.3%) and 14(23.3%) had post-operative seizures and recurrence of hematoma respectively. There was no significant association between type of burr-hole and hospital stay (p 0-884), seizures (p 0.448) or recurrence (p 0.542). Hospital stay (p<0.000) and seizures (p-0.005) were inversely proportional to GCS scores on presentation. Recurrence rates were not affected by age (p-0 .175) or gender (p-0 .281).

**Conclusion:**

There was no significant difference between outcomes of single and double burr-hole surgeries; the former must be preferred because of lesser iatrogenic trauma. GCS-score on presentation was validated as a negative association to anticipate post-operative outcomes.

## INTRODUCTION

Subdural hematoma (SDH) is accumulation of blood between dura-mater and brain surface. Chronic subdural hematoma (CSDH) is one of the most frequently encountered malady in neurosurgical clinics.^1^ It is generally considered as benign and easily treatable problem with an increasing incidence with age.^2^-^4^ If not timely diagnosed or treated, it carries significant morbidity or mortality and vice versa; and is also a treatable cause of stroke and dementia in peculiar groups. Majority being post-traumatic, often following minor head trauma,^4^ rest are mostly preceded by coagulation defects, intracranial hypotension, repeated intra cranial hemorrhage, increased outer membrane exudates, or occlusion of cerebrospinal fluid.^1^ Administration of anti-coagulants/antithrombotic drugs has been established as a vital iatrogenic factor.^5^ It develops subtly over days or weeks, and may remain undiagnosed for even months or even a year. Clinical presentation may vary from no symptoms to unconsciousness.^1^ Most patients present with GCS score 12 or more and without loss of consciousness.^6^ Clinical outcomes are inversely proportional to neurological status at presentation;^3^,^7^ GCS lesser than 8 anticipates poor prognosis. It is a frequently missed clinical entity; those with modest symptoms (headaches, memory disorders, balance disorders, cognitive disorders etc.) are often misinterpreted as signs of dementia, circulatory failure-basilar vertebra or Alzheimer’s disease.^4^ The role of apt clinical assessment and radiological imaging to rule out CSDH cannot be overemphasized. Diagnosis depends upon size of lesion and hematoma’s intima, history of fresh hemorrhage/hemolysis, and any associated primary or metastatic dural disease. Simple radiological scans like cerebral CT scans often highlight these hematomas; while early neurosurgical intervention validate favorable prognosis.^1^,^4^ When the cause is unknown, the color of the hematoma on neuroimaging study can hint towards duration of lesion. There may be hypo-dense chronic lesions, or mixed density images of acute on chronic bleed.

These slowly progressive bleeds may stop without considerable damage. Literature revealed that only 22% of patients with chronic subdural hemorrhage had outcomes worse than “good” or “completely recovered”.^8^ Timely and optimum management averts grave complications.^3^ Treatment is aimed to return every patient to previous daily life, and is often achievable.^4^ A multitude of surgical drainage procedures are available but clear-cut guidelines are jeopardized and scanty. Single or double burr hole drainage is the first-line therapy, being most effective and frequently utilized, with minimal co-morbidities, complications and recurrence.^1^,^2^ Nonsurgical management is reserved for asymptomatic or patients at high risk for operation.^1^ Burr-hole craniostomy (also known as trepanning, trepanation, trephination, trephining) is a time-tested intervention in which a hole is drilled or scraped into the human skull, exposing the dura-mater to release pressured blood build-up from an injury or manage issues pertinent to intracranial diseases.^9^ Various international researchers have compared the single-hole and double-hole drilling, but unfortunately reported follow-up periods are short-lived, often limited to acute hospitalization.^3^ Although neurosurgical clinics in Pakistan frequently manage clients of CSDH, nevertheless, formal studies are skimpy and no such study has yet been conducted at subject hospital. This is the pioneer survey of its type which aimed to compare outcomes of two types of burr-hole surgeries in terms of hospital stay, seizures or recurrence; simultaneously keeping in mind age, gender, site of hematoma, coagulation profile and GCS-scores at presentation. Sub-dural drainage and irrigation was administered to all patients because of their validated outcomes. Questions posed were, is there any difference in outcomes of two types of burr-hole drainage for CSDH and what the significant prognostic associations are. The research is anticipated to generate interesting results and provide with a way forward to scientists for conduction of better researches in similar populations with expanded sample and extended variables.

## METHODS

This prospective cohort study was carried out in department of neurosurgery, under the kind auspices of Combined Military Hospital, Multan, from December 2016 to August 2018; preceded by formal approval by Ethical Review Board, and written informed consents by patients (or wards of comatose personnel). Patients were allocated into two groups by randomized control trial; 30 persons in each group. Symptomatic adults with diagnosed chronic subdural hematoma via CT/MRI scans were included, while those < 18 years age, already operated for CSDH, with post CSF diversion CSDH or with prior history of seizures, coagulopathies or intake of anti-coagulants were excluded by non-probability purposive sampling. Patients who lost post-operative follow-up were replaced by other eligible cases.

Group-A and Group-B underwent single and double burr holes drainage respectively (all with sub-dural drain and irrigation). Detailed management encompassed optimum history and examination, Glasgow coma scale on presentation, side of hematoma, hospital stay, coagulation profile, postoperative seizures, recurrence of hematoma, laboratory investigations and imaging studies. Coagulation parameters included platelet count, prothrombin time (PT) and activated partial thromboplastin time (APTT). At the time of imaging, CSDH was analysed as low density or mixed, based on density of hematoma relative to brain tissue. Patients with GCS 8 or less were operated under local anesthesia; rest under general anesthesia. After surgery, depending upon neurological status, they were nursed in intensive care unit or neurosurgery room. Stable patients were then discharged. Any evidence that followed them for a month showed signs of relapse and regeneration. Data analysed via SPSS-21, qualitative expressed as frequency and percentage, summed-up as mean±standard deviation (range minimum-maximum). Dependent variables (hospital stay, recurrence and seizures) were cross-tabulated with independent variables via Chi-square; p-value <0.05 was taken as significant.

## RESULTS

**Fig. 1:**
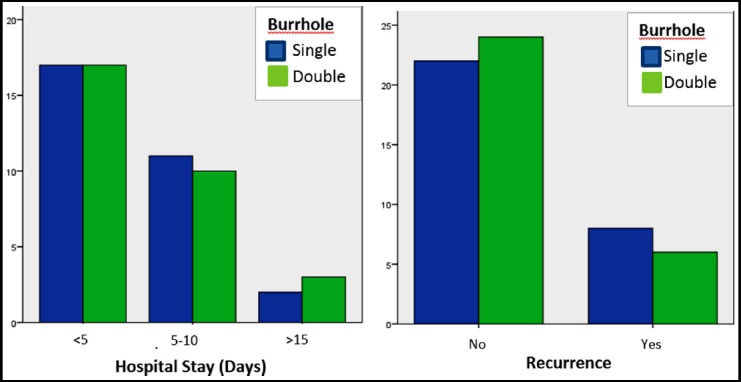
There was no significant impact of type of surgery on hospital stay or recurrence rate.

**Fig. 2:**
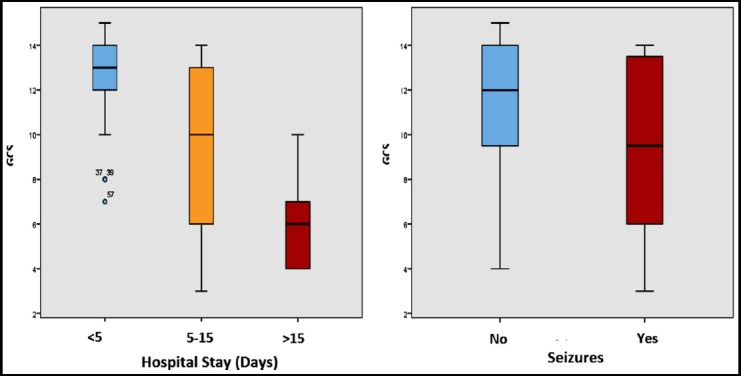
GCS-scores at presentation inversely affected hospital stay and occurrence of seizures.

Age and GCS scores were 62±13.694 (range 38-94) and 11.00±3.350 (range 3-15) respectively, males being 40(66.7%). In hospital stay of patients was as follows; <5 days 34(56.7), 5-15 days 21(35.0) and >15 days 5(8.3). Post-operative fatality was Nil, while 8 (13.3%) and 14 (23.3%) had post-operative seizures and recurrence of hematoma respectively. Independent and outcome variables along with their mutual associations are illustrated in Table-I. There was no significant difference between type of burr-hole and hospital stay (p-0.884), recurrence of hematoma (p-0.542) or seizures (p-0.448) Fig.1 while hospital stay (p<0.000) and seizures (p-0.005) were inversely proportional to GCS scores on presentation (Fig.2). Subjects with bilateral hematoma significantly suffered more with seizures (p-0.001). Age and gender remained more inert to cast significant impact on any outcome.

**Table I:** Relationship between independent and outcome variables (N-60).

Variable	Frequency (Percent)	Hospital Stay (Days)			Recurrence		Seizures	

		<5	5-15	>15	No	Yes	No	Yes
Age (Years)		p-0.904			p-0.175		p-0.662	
<40	1(1.7)	1	0	0	0	1	1	0
40-60	30(50.0)	16	11	3	24	6	27	3
>60	29(48.3)	17	10	2	22	7	24	5
Gender		p-0.432			p-0.281		p-0.788	
Male	40(66.7)	25	12	3	29	11	35	5
Female	20(33.3)	9	9	2	17	3	17	3
GCS-Scores		p-0.000			p-0.211		p-0.005	
3-8	14(23.3)	2	8	4	9	5	9	5
9-15	46(76.7)	32	13	1	37	9	43	3
Hematoma		p-0.898			p-0.879		p-0.001	
Right	29(48.3)	18	9	2	23	6	26	3
Left	24(40.0)	13	9	2	18	6	23	1
Bilateral	7(11.7)	3	3	1	5	2	3	4
Coagulation Profile	p-0.957	p-0.145			p-0.499			
Normal	47(78.3)	27	16	4	38	9	40	7
Deranged	13(21.7)	7	5	1	8	5	12	1
Burr-hole		p-0.884			p-0.542		p-0.448	
Single	30(50.0)	17	11	2	22	8	27	3
Double	30(50.0)	17	10	3	24	6	25	5

## DISCUSSION

Neurosurgeons quite frequently deal with chronic subdural hematomas.^10^,^11^ Acute hematomas are typically preceded by severe trauma, chronic collections often herald minor trauma or non-traumatic causes.^5^,^12^ CSDH is an encapsulated collection of blood and fluid on the surface of the brain following an intracranial hemorrhage,^5^,^12^ where low pressure venous hemorrhage from bridge vein strips the arachnoid from dura. Small subdural hematomas may occur repeatedly and absorbed spontaneously. A large amount of subdural blood usually organizes and forms a vascular membrane surrounding the hematoma. Repeated bleeds in small and friable vessels within these membranes may be responsible for the growth of chronic subdural hematomas. Ishfaq A. et al.^12^ and Edlmann E et al.^13^ have described the pathophysiological development of CSDH in depth; The surrounding membrane of clot is a source of fluid exudation and hemorrhage, angiogenic stimuli cause formation of fragile blood vessels within membrane walls, whilst clot formation is prevented by fibrinolytic agents with resultant continued hemorrhage. An inflammatory cascade is triggered by inflammatory cells/markers, while fibroblasts invade the clot to form cortical and dural neo-membranes. The liquefied clot and fibrin-degradation products further incorporate into new clot. Fluid resorption or re-bleed define the further course of disease. Few hematomas may also expand due to osmotic effects or varied calcification mechanisms.^14^ Brain damage is caused by direct pressure, increased intracranial pressure (ICP) or associated parenchymal damage.

The gradual rise in incidence of CSDH is expected to persist, therefore, it’s imperative to pin-point definitive and more refined operative regimes.^5^,^15^ Various surgical manoeuvres are available, nevertheless, optimal technique remains controversial.^10^-^12^,^14^,^16^-^18^ Less invasive procedures like twist-drill craniostomy (TDC) and burr-hole drainage(BHD) often gain priority;^1^,^2^,^13^,^16^-^18^ former being more safe and effective method for maximal thickness areas, or for bedside drainage of patients at high risk for surgery.^15^,^16^,^19^

In this research, operators did not find any significant difference in results of single or double burr-hole surgery in terms of hospital stay, seizures or hematoma recurrence. Various scientists have documented that type of intervention or number of burr-holes do not alter the outcomes significantly.^3^,^7^,^18^,^20^,^21^ Few scientists proclaimed superiority of single burr-hole in terms of complications and recurrence rates;^22^ others categorized double burr-hole as more effective.^10^

Right-sided, left-sided and bilateral hematoma were found in 48.3%, 40.0% and 11.7% clients of this study respectively, and all patients received irrigation. FARHAT NETO J. et al reported right CSDH in 41%, left in 43% and bilateral in 16% patients.^14^ Literature concurred lesser recurrence with irrigation,^10^,^11^,^18^,^21^ although death rates or other outcomes remained almost the same,^11^ few refuted any such associations.^7^ Subjects with better GCS-scores depicted shorter hospital stay span and lesser seizures. Lower GCS antedate poorer prognosis.^1^,^4^

The post-operative mortality rate was nil. In hospital mortality rate of 1.4% has been reported,^15^ with much higher rates after one year.^3^ In this study, 23.3% were agonized by recurrence, with no significant difference among various age groups; these results were comparable to 20-22% recurrence rates in other literature, where elderly suffered more.^2^,^15^

Authors could not evade all limitations. Due to incomplete coverage, generalization of study has to be done with caution. Comparison with other surgical procedures and incorporation of multiple hospitals could have rendered varied results. Nevertheless, it was first of its type to be conducted in a metropolitan city of Pakistan, a developing country. Burr-hole surgery was selected as it is the commonest drainage procedure adopted for CSDH in subject hospital. Type of operation was compared with various demographic, pre and post-operative variables to analyse validity of both procedures. This study is opined to have a high predictive value pertaining surgical outcomes and prognosis of adults suffering from CSDH who undergo single or double burr-hole drainage.

## CONCLUSION

Administration of single or double burr-hole technique did not significantly alter surgical outcomes for patients of CSDH; former must be preferred because of lesser iatrogenic trauma. GCS-score on presentation can anticipate post-operative outcomes due to its negative association with various prognostic factors.

### Authors’ Contribution

**HUK & KA** conceived & designed the study.

**HUK** collected data.

**GHT** analysed and inferred data.

**KA & GHT** endorsed the manuscript.

**HUK** finally approved the manuscript.

**KA** takes the responsibility and is accountable for all aspects of the work in ensuring that questions related to the accuracy or integrity of any part of the work are appropriately investigated and resolved.
